# Testing the Labeling Effect in Autistic Children

**DOI:** 10.1007/s10803-024-06388-1

**Published:** 2024-05-27

**Authors:** Sergio Parrillas-Manchón, Elena Castroviejo, José V. Hernández-Conde, Ekaine Rodríguez-Armendariz, Agustín Vicente

**Affiliations:** 1 Department of Linguistics and Basque Studies, University of the Basque Country (UPV/EHU), Vitoria-Gasteiz, Spain; 2 Universidad de Valladolid, Valladolid, Spain; 3 Ikerbasque-Basque Foundation for Science & Department of Linguistics and Basque Studies, University of the Basque Country (UPV/EHU), Vitoria-Gasteiz, Spain

**Keywords:** Linguistic categories, Concept acquisition, Inference, Labeling

## Abstract

**Purpose:**

Our objective was to test the labeling effect in autistic children. The effect has been robustly tested in typically developing (TD) individuals. TD children expect that any two objects that receive the same linguistic label will have similar properties, which suggests that they generate concepts based on acts of labeling. The labeling effect has not been tested on autistic children, who may not be equally attuned to the relevance of linguistic clues or may not generalize as swiftly as TD children.

**Methods:**

We reproduced Graham et al.,’s (Frontiers in Psychology 10.3389/fpsyg.2012.00586, 2013) design on 30 autistic children of different ages. Participants were divided into two groups depending on whether objects presented to them were named alike or differently (Same or Distinct Label between-individuals condition). The dependent variable was the number of target actions the child performed on an object, depending on whether that object made the same sound as a previously shown test object.

**Results:**

We did not reproduce results similar to those reported in Graham et al., (Frontiers in Psychology 10.3389/fpsyg.2012.00586, 2013). Children in the Same Label group did not perform significantly more actions than children in the Distinct Label group when the objects that were handed to the children did not make the same sound as the test object.

**Conclusions:**

Autistic children do not seem to be sensitive to the labeling effect to the same extent as TD children. If these results are confirmed, intervention programs for autistic children should consider trainings on this way of generating concepts shared by their linguistic community.

The goal of this paper is to discuss the sensitivity of the so-called labeling effect in autistic children. While it has been observed that naming two or more objects with the same label creates some expectations in typically developing children regarding hidden or non-obvious properties these objects may share, it is still to be studied whether autistic children use linguistic cues in the same manner. The labeling effect has been linked to the communicator’s willingness to share a common categorization with their interlocutor on the basis of linguistic labels. Also, the labeling effect involves generalization on the basis of label sharing. Given the observed different social and inferential profiles in typical and atypical development, we hypothesized potential differences in the sensitivity to the labeling effect.

In order to obtain novel data on this research question, we followed an experimental design by Graham et al., (2013), this time administered to a group of autistic children whose ages range from 3 to 9 years. While autistic children engaged in the activity proposed by the study, we failed to reproduce the results reported by Graham et al. with neurotypical children, which suggests that autistic children may exhibit difficulties in understanding labeling in the way typically developing (TD) children understand it.

In the remainder of this paper, we provide some background on the labeling effect and the experimental setting the current experiment is based on. We subsequently lay out the details of the design we administered and discuss the collected data.

## Background

### The Labeling Effect

The labeling effect is a well-attested phenomenon in typical development. Beginning with 10 months, and at least until they are 11 years old, children presented with two or more objects that receive the same label expect that these objects share the same non-obvious, or hidden, properties (e.g., a sound that one of the objects makes). Thus, if children are introduced to two novel objects, both of which are called *blicket* by the experimenter, and one of them rings when pressed, children expect that the other object will also ring if pressed. This is taken to show that children grasp that two or more objects that receive the same name belong to the same category and that they group such objects together under the same mental representation (Waxman & Braun, 2005; Waxman & Markow, 1995). Only when two objects are conceptualized as belonging to the same category do subjects expect that they will share non-obvious properties (e.g., only when two animals are conceptualized as belonging to the same category do subjects expect that if one of them barks, the other one will also bark).^1^ The expectation generated by label sharing can even make children revise previous expectations based on perceptual similarity. In principle, if children are shown three objects: *a, b,* and *c*, with *a* and *b* being more similar than *a* and *c*, and they see *a* making a sound*,* they will expect that *b*, and not *c*, will make the sound. However, if *a* and *c* receive the same name, and *a* makes a sound, children will expect that *c*, and not *b*, will make the sound. The opposite expectation arises when objects receive distinct labels: children expect differently named objects to have different non-obvious properties (Dewar & Xu, 2009; Graham et al., 2013).

In sum, in the absence of any linguistic clue, children categorize by perceptual similarity. Linguistic labels, however, induce children to generate concepts aligned with linguistic categories. Therefore, the labeling effect refers to the impact that linguistic labels have in generating concepts that override perceptually based categorizations of the world (Dewar & Xu, 2009; Plunkett et al., 2008; Waxman & Markow, 1995; Welder & Graham, 2001; Westermann & Mareschal, 2014, among many others).

The labeling effect has been robustly tested using different paradigms in TD children of different ages and cultures (Long et al., 2012), beginning at nine (Dewar & Xu, 2007) or 10 months (Dewar & Xu, 2009), in preadolescents (Sloutsky & Fisher, 2012; Sloutsky & Lo, 1999), and adults (Lupyan et al., 2007). There has been some discussion about how and why labels have such an effect in cognition.^2^ Thus, Waxman and Markow (1995) held that the use of labels guides infants’ attention to less noticed perceptual similarities between objects, restructuring an unsupervised similarity metric used for categorization. That is, the use of a label can make children look for commonalities that are not as salient as purely perceptually based commonalities. For instance, TD children are known to generalize on the basis of shape (what is known as a shape-bias generalization: (Smith, 1999). The use of labels can override such a bias by directing children’s attention to other features of objects and grounding their generalizations based on such features. Ferguson & Waxman (2017, p. 527) suggest that the cognitive effect of labeling relates to the linguistic label’s “status as a social signal”, thus gesturing towards a communicative approach to the labeling effect. Such a suggestion is supported by results showing that linguistic labels have an effect in categorization that no other sound signal has (Ferry et al., 2010), which indicates that words as public signs have a special status in category formation. Henningsen-Schomers et al., (2022) report that supplying a verbal label during concept acquisition helps form and consolidate neuronal ensembles for specific concepts and meanings. That is, verbal labeling would not just reorganize representations of categories, but also help consolidate them in memory.

To date, the labeling effect has not been tested on autistic children. However, there is reason to believe that autistic children may not exhibit the same sensitivity to linguistic labels that TD children exhibit. As just suggested, part of the reason why the effect is so robust in TD children may be that TD children assume that labels are not arbitrary but have been introduced by others who are trying to communicate information about what objects belong to a certain category. Autistic children may not be aware of this role of linguistic labels, given their difficulties in the socio-communicative domain (American Psychiatric Association [APA], 2022). In particular, they may exhibit difficulties understanding the complexities of the act of naming. When experimenters label two objects with the same name, TD children seem to understand that these two objects belong to the same category of objects just because experimenters have indirectly told them that this is the case. Autistic children may not recognize that giving the same name to two objects has the implications that it has for TD children.^3^

On the other hand, TD children seem to exhibit a considerable flexibility when they revise their perceptually based categorizations, adopting a linguistic-based categorization. It is well known that autistic children have more difficulties than TD children with regards to induction and generalization (Johnson & Rakison, 2006; Klinger & Dawson, 2001; Rutherford & McIntosh, 2007). For instance, some studies have found that the shape bias appears later in autistic development, such that autistic children build narrower categories than TD children of the same age (Hartley & Allen, 2014; see also Floyd et al., 2021). Thus, autistic children may have more difficulties generating categories based on linguistic stimuli alone, as well as generalizing from the observed behavior of one object to the behavior of another that only shares a label with the first one. On the other hand, as mentioned, adopting a label-based categorization implies some degree of flexibility that according to many studies is also difficult in autism (Landry & Al-Taie, 2016).

We consider that testing the labeling effect on autistic children is of great relevance. If, as Waxman & Markow (1995) suggest (see also Waxman & Braun, 2005), labels are invitations to create conceptual categories, a diminished or delayed sensitivity to labeling might involve that autistic children experience difficulties generating the categories that their TD peers generate. Also, a diminished or delayed sensitivity to the labeling effect may involve that concepts themselves may be more volatile than in the neurotypical case.^4^ With the aim of testing the labeling effect in autistic children for the first time, we made use of an experimental design by Graham et al. (2013) with TD children, in which children were invited to manipulate an object after being presented with another one that made a specific sound. This kind of action-oriented paradigm seems appropriate to gauge autistic children’s reactions. It is also adequate to test how autistic children—especially those with poorer linguistic abilities—establish label-object pairings, since it has been claimed that, in some cases, children who learn labels for 2D representations may fail to apply them to real world objects (Preissler, 2008). Since we were interested in studying the labeling effect on objects, we preferred not to use pictures or photographs in a computer screen, but actual objects. Moreover, the administration of Graham et al.’s design was relatively quick. From another action-oriented study on word learning by exclusion, we also knew that the participants in our study were keen to take part in a study like this. We thus concluded that Graham et al.’s paradigm was especially suitable for assessing the labeling effect in our population of interest.

Let us now turn to providing the necessary specifics of Graham et al.’s experiment, so we can lay out the details of the experiment put forth in the present paper.

### Graham et al.,’s (2013)* Experiment*

Graham et al. assessed the labeling effect by observing the manipulation of objects by 15-month-olds. In the experiments, there were two sets of objects: target objects had a non-obvious property (e.g., could make a sound if shaken, but this was not evident at a glance), and test objects varied in shape similarity with respect to target objects. In this design, the behavior of two groups of children was compared. The Same Label group was presented the test objects with the same label as target objects, while the Distinct Label group was presented the test objects with a different label from the target objects.

The hypothesis in Graham et al.’s study was that children in the Same Label group would create a common category for the pair of objects to which the same label was assigned. In contrast, the children in the Distinct Label group would not establish a common category for the pair of objects with different names, so there would be no generalization of the non-obvious property from target to test objects. The procedure employed to test whether children were generalizing the non-obvious property consisted in counting the total number of imitated actions on the test object (e.g., shaking events) at each different condition, under the assumption that children would make more actions on test objects when they expected to share the same non-obvious property with the target object. If children expected the test object to produce the same sound as the target object, but it did not, they would persist acting on the object for a longer time than if they did not expect the test object to make the same sound as the target object.

**Fig. 1:**
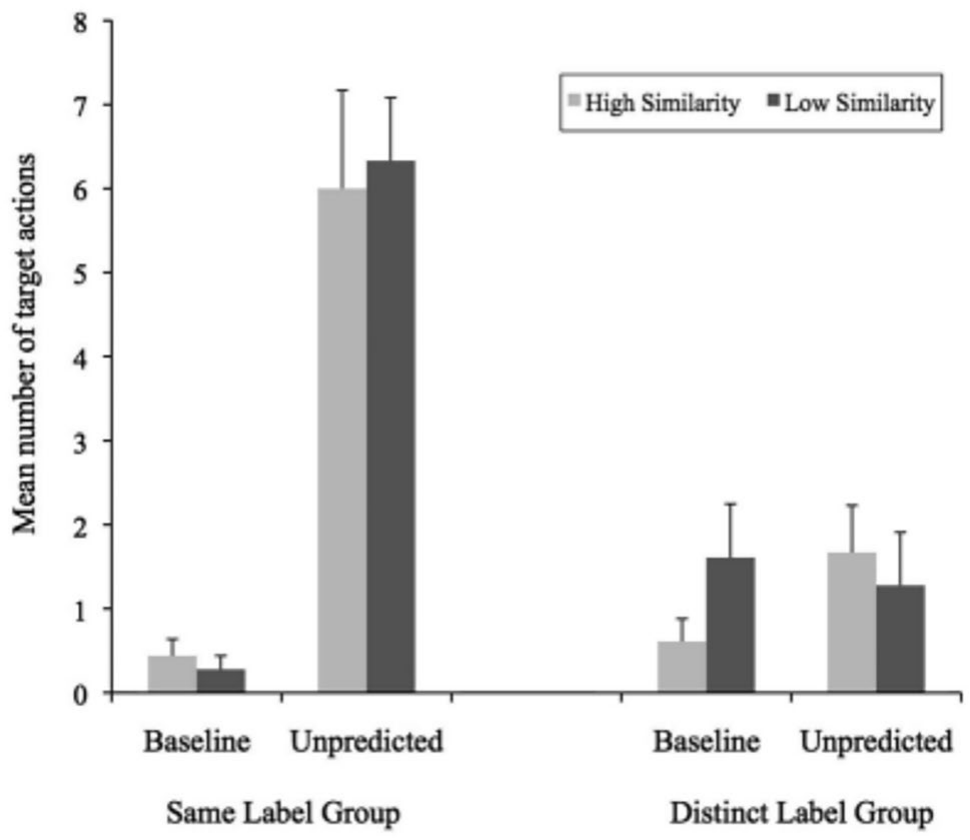
Mean number of target actions performed on test objects as a function of label group, condition, and similarity (Graham et al., 2013)

The results of Graham et al.’s experiment seemed to confirm the labeling effect at 15 months of age. Indeed, there was a significant difference between the mean number of actions on the test object by the children in the Same Label group and the mean number of actions in the Distinct Label group (see Fig. 1).^5^

## Methods

### Research Question and Hypothesis

The research question put forth in this paper is whether or not autistic children exhibit the same sensitivity to the labeling effect as TD children have shown to exhibit in works such as Graham et al., (2013). More specifically, the study reported in this paper wanted to test whether there would be a significant difference between the number of actions performed on the test object by the children in the Same Label group and by the children in the Distinct Label group when presented a test object that did not make a sound. For the reasons exposed above, we hypothesized that there may be no such difference, since autistic children may not exhibit the same sensitivity to the labeling effect as TD children.^6^

### Participants

Thirty autistic Spanish-speaking children participated, eight girls and 22 boys. All of them had been administered the ADOS-2 test (Lord et al., 2012, Spanish edition) and their outcome was at least above the threshold for autism spectrum conditions. The 30 children were divided into two groups, following the procedure of Graham et al. (2013): Same Label and Distinct Label, as shown in Table 1.

**Table 1:** Characteristics of participants by Group (Before exclusion of outliers)

	Same label	Distinct label
N	13	15
Chronological Age	59.5 (SD 13.7)	69.4 (SD 19.1)
Autism severity	5.6 (SD 1.2)	5.7 (SD 1.8)
Verbal Mental Age	37.4 (SD 13.3)	49.9 (SD 25.2)
Non-Verbal IQ	92.5 (SD 12.6)	100.8 (SD 14.4)

Verbal Mental Age was obtained through the administration of the PPVT-III (Dunn et al., 2010) and Non-Verbal IQ, through the administration of the Leiter-3 scale (Koch et al., 2019)

In the Same Label group there were children between two years and nine months (2;9) and six years of age (6;0), with a mean of four years and eight months (4;8). In the Distinct Label group, the age range was between three years and nine years, with a mean of 5;9. None of the participants were users of augmentative and alternative communication systems. All of them had therapeutical intervention at the local Early Attention Service twice a week (half an hour per session). Both groups were similar with respect to autism severity mean scores (a mean of 5.7 severity in both groups on a scale from one to 10 and threshold for autism spectrum at four, according to Gotham et al.,'s (2009) calibration of ADOS raw totals across modules). We opted to match both groups on autism severity because our main hypothesis (i.e., that autistic children may not be as sensitive to the labeling effect as TD children are) relates to autistic features. On the other hand, as said before, in typical development the labeling effect is robust from at least nine months (Dewar & Xu, 2007) or 10 months (Dewar & Xu, 2009) at least to preadolescence (Sloutsky & Lo, 1999; see Lupyan et al., 2007 for similar results with adults). This means that it is robust regardless not only of chronological age but also of verbal mental age (at least in the range of chronological and verbal ages that participants in the current study had). While we cannot discard that chronological or verbal mental age may have a different impact in the autistic population than in TD—which suggests a possible limitation of the study —, we thought more appropriate to use severity measures as a way to establish the groupings.

All children went through a warm-up phase where the experimenter manipulated three familiar objects that then handed to the children while inviting them to perform the same action. This warm-up phase served to introduce children to the imitation task. All children who participated in the study displayed imitation in the warm-up phase.^7^ Only two children had to be excluded due to lack of attention and restlessness.

### Materials

**Fig. 2:**
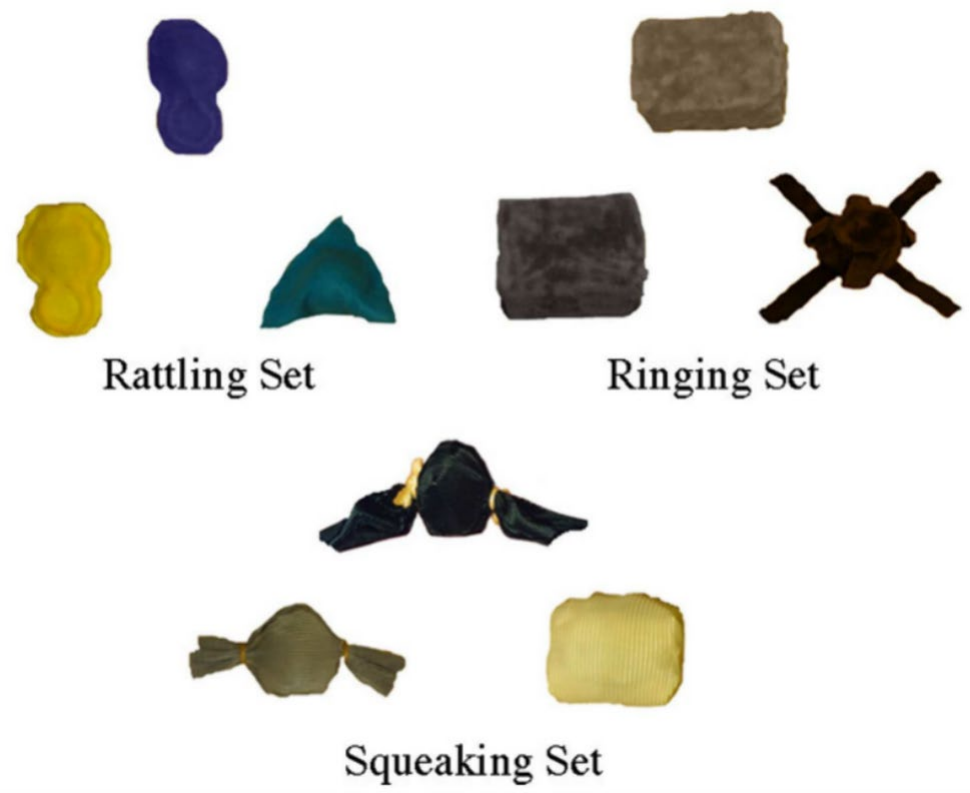
The three sets of objects in Graham et al., (2013): the rattling set, the ringing set, and the squeaking set

**Fig. 3:**
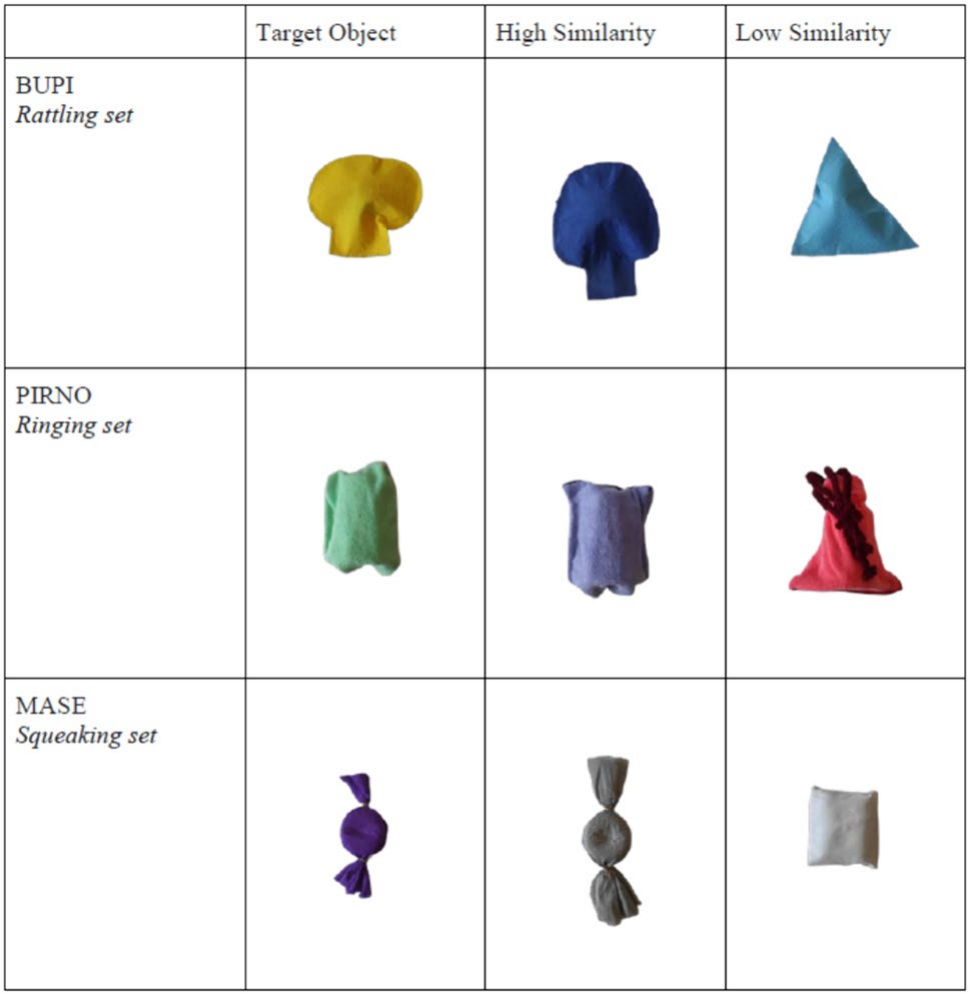
The three sets of objects in our experiment: rattling set, ringing set and squeaking set. Within each set, there was a target object, a High-similarity test object and a Low-similarity test object

This experiment used three sets of materials with a slightly different appearance than Graham et al.,’s (2013), but which were defined in the same way by the type of sound they made: rattling set, ringing set, and squeaking set (see Fig. 2). The names assigned to the target object of each set were *bupi*, *pirno,* and *mase*. These met the conditions of being pseudowords that follow the typical phonotactic distribution of the Spanish language. Likewise, the objects were different depending on the similarity variable (with respect to the prototype), which had two levels: High and Low. This is shown in Fig. 3.

### Design

Following Graham et al.,'s (2013) design, aside from the between-individuals variable (Same vs. Distinct Label), there were two within-individual variables. First, degree of similarity (High vs. Low), as depicted in Fig. 3; second, predictability of the outcome (Predicted, Unpredicted, Baseline). Let us clarify what these three latter conditions consisted of. Focusing on the set of objects that made a sound if shaken, in the Predicted condition, the experimenter would shake the target object, thus making the sound, and then would give the child a test object that would make the same sound as the target object if shaken. In the Unpredicted condition, the experimenter would shake the target object, and then hand a test object that did not make any sound. Finally, in the Baseline condition, the experimenter would not manipulate the target object, so that the child would not be compelled to make any specific action on the test object.

Putting it in more simple terms: children saw what actions the experimenter performed on the target object and were handed a test object that could be very similar or quite dissimilar to the target object. In the Predicted condition, the test object made the same sound as the target object when the action of the experimenter was imitated. In the Unpredicted condition, the test object was disabled, and so no sound was emitted, no matter how many times children imitated the experimenter's actions. The main prediction of the study is that children in the Same Label group would produce more actions on the test object than children in the Distinct Label group in the Unpredicted condition. The rationale is that children in the Same Label group would expect that two objects named alike would have the same sound properties, whereas children in the Distinct Label group would not form such an expectation. The experimental session was developed through six trials. Target objects appeared twice: the first time with their High similarity pair and the second time with their Low similarity pair, or vice versa.

**Fig. 4:**
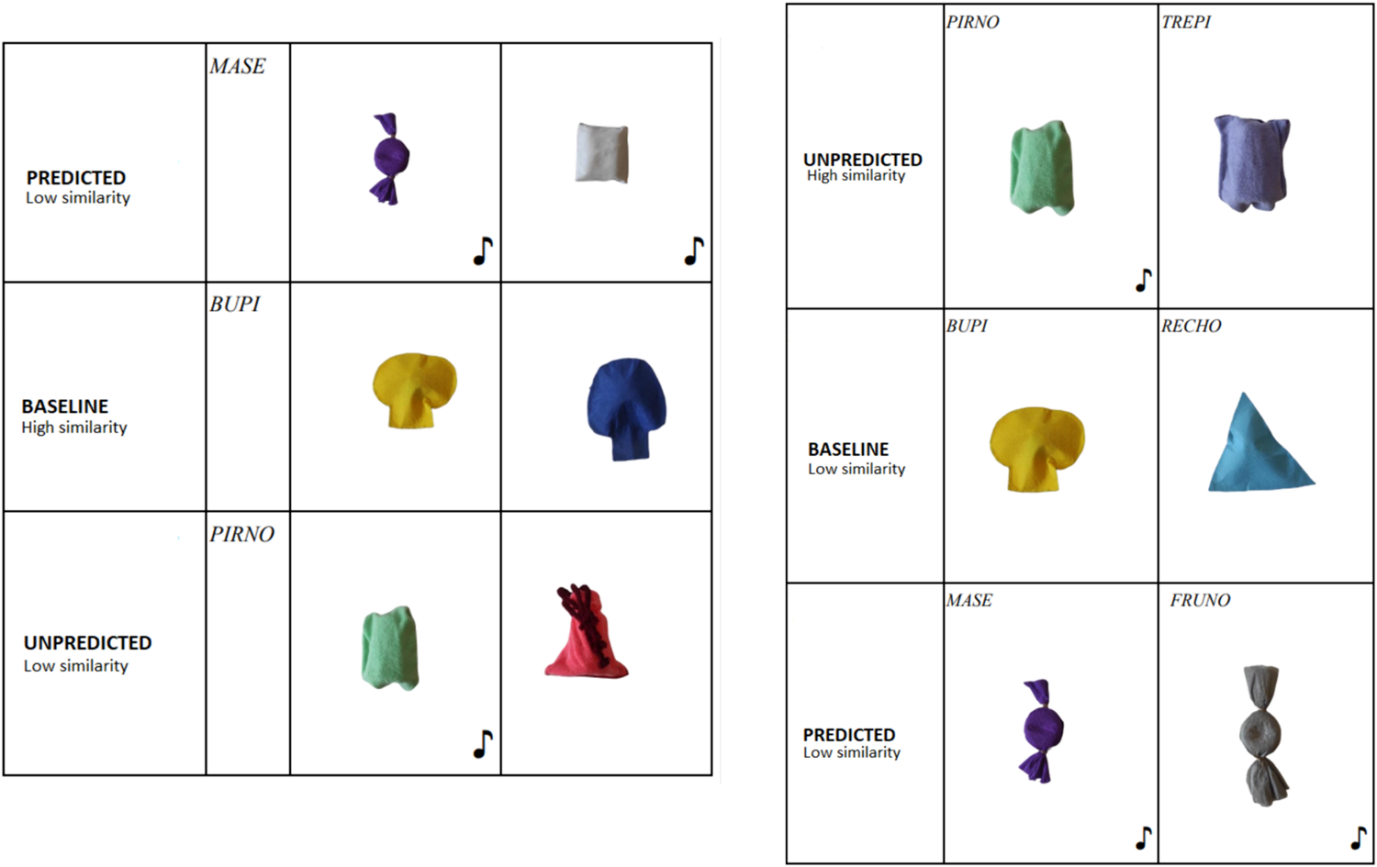
Three trials, half session in the DL group (left) /// Three trials, half session in the SL group (right). Between groups there is an inversion of sound distribution in both predicted and unpredicted conditions

For each object, there was a disabled version and a sounding version. As mentioned above, there were three possible combinations of objects (both without sound, only one with sound, or both with sound). These three combinations were assigned the Predicted, Unpredicted, or Baseline condition. This is shown in Fig. 4 below.

The presentation of the materials was counterbalanced. For this purpose, between High-similarity and Low-similarity Predicted and Unpredicted conditions there was a set change. We generated different combinations in the order of appearance of the set per condition.

The novel objects that were used, as well as the novel labels that were assigned to them, were created specifically for the experiment with the aim that the encyclopedic knowledge of each participant did not bias their actions.

### Procedure

All experimental sessions were recorded with a camera, as detailed in the informed consent that families had to sign before starting the experiment. Children were also consulted about their willingness to participate in the experiment. Parents remained in the room throughout the session but did not come into contact with their children and did not intervene unless the child needed to be assisted, as required by our ethical commitment ratified by the Ethics Committee for Research involving Human Beings from [Omitted for blind review]. The recordings were essential for data extraction, and we acted under the requirements of Organic Law 3/2018, of December 5, Protection of Personal Data and guarantee of digital rights (LOPD-GDD).

The experimenter was an experienced occupational therapist who works with autistic children in a clinical setting. She was familiar to most children and families, as she had administered the ADOS-2 test to most of the participants in the study. In the experiment, she was assisted by the first author of the paper. The experiment was administered in a quiet room in the Research Centre where the authors work, which was also familiar to the participants. The experimenter was instructed to stop at any sign of discomfort she could perceive in the participants or their families.

The six trials of each experimental session followed a rigorous script recreated through the explanations offered by Graham et al., (2013) in this regard.

#### Warm-up

The children were shown three objects with which they were familiar (a fan, a toy wheel, and a toy hammer) so that they got used to the imitation dynamic. This phase was common in the Same Label and Distinct Label groups.

#### Experimental Phase

In the first step of the experiment, the experimenter would take the target object from any set and show it to the child at a distance. The experimenter would repeat five times “Look! Look at this! Look! This is a *mase*”. Only within the Predicted and Unpredicted conditions did the experimenter perform a specific action on the target object five times. Then, in all conditions, the target object would be given to the children for them to explore it for 10 s, being afterwards removed. The target object would be left in plain sight, but out of reach. In the second step, the experimenter would pick up the test object, which could be a High similarity or Low similarity item. She would then say five times “Look! Look at this! Look! This is a *mase*” to the children in the Same Label group, and “Look! Look at this! Look! This is a *fruno*. This is not a *mase*, it is a *fruno*!” to the children in the Distinct Label group. After that, the experimenter would hand the test object without performing any actions on it before giving the object to the child. The child was allowed to explore the test object for 20 s.

### Data coding

The dependent variable in this experiment was the number of actions that the child imitated on the test object after having seen and possibly reproduced them on the target object. As we have explained above, Graham et al., (2013) considered that what allows us to observe the labeling effect is the comparison of the number of actions performed on the test object between Same Label and Distinct Label in the Unpredicted conditions.^8^

The coding of the actions followed rigorous guidelines, taken from Graham et al., (2013). Actions that deviated from the action modeled by the experimenter were excluded: blows against other objects, against the table, throws, etc. In the Predicted and Unpredicted conditions, only the actions that successfully imitated the actions of the experimenter were counted. If the child performed the action with two hands, even if one hand reached its destination shortly before the other, it had to be counted as a single movement. The back and forth that occurred in the swaying of an object counted as a single action. Accidental production of the sound was not counted. On a spreadsheet, each coder wrote down the number of target actions on the test object for each of the six trials each child participated in (Baseline, Predicted, and Unpredicted conditions by High and Low similarity conditions). The coding of the number of actions of the recorded experimental sessions was conducted, first, individually by three of the co-authors of this work. We used the irr package in R (Gamer et al., 2019) to calculate the Interclass Correlation Coefficient (one way model) for consistency among three raters who rated 165 cases. The ICC value was 0.729 (95% CI 0.666, 0.784), indicating relatively high reliability among the raters. The coders subsequently met to discuss a few problematic cases; to address them, they revised the videos several times at different playback speeds, until an agreement was reached.

## Results

### Descriptive Statistics

**Fig. 5:**
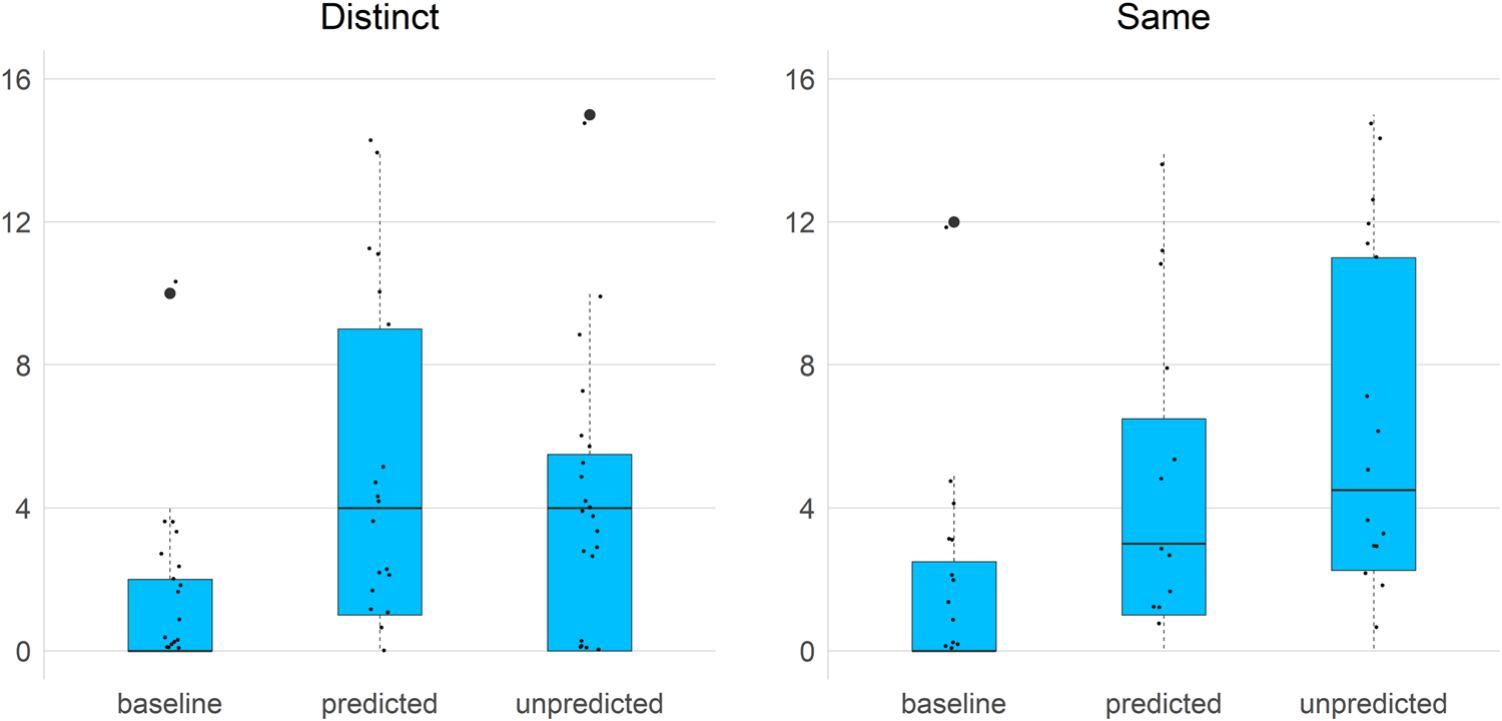
Number of target actions on test object by label and predictability conditions

In Table 2 and Fig. 5 we can explore the descriptive statistics showing the distribution of the dependent variable by predictability condition and label.

**Table 2:** Mean number of target actions on test object by predictability condition and label

	Same label	Distinct label
Baseline	3.32 (SD 4.96)	4.53 (SD 8.39)
Predicted	10.96 (SD 10.81)	10.30 (SD 11.10)
Unpredicted	8.08 (SD 9.42)	7.07 (SD 8.10)

Before exclusion of outliers

The box plot represents median (horizontal lines), interquartile range (blue square) and highest and lowest sample (whiskers). The above data suggests a relevant difference between the Unpredicted condition in the Same Label group vs. the Unpredicted condition in the Distinct Label group, both between the two means (Table 2) and the spread of the dependent variable (Fig. 5), to be corroborated below. In particular, there is a higher mean number of actions on the test object in the Unpredicted condition in the Same Label group than in the Unpredicted condition in the Distinct Label group.

### Exclusion of Outliers

Some observations are in order regarding the possible treatment of outliers. In the experimental literature, depending on the number of deviations from the mean—one, two, or three —, three criteria can be applied. Graham et al. used the three-sigma rule as a criterion (Graham et al., 2013, p. 4), that is, they excluded those children whose performance was above or beyond the mean by three standard deviations. A more cautious heuristic implies employing a more stringent rule (i.e., two-sigma rule) in order to reduce the impact of the more extreme values. In our case, we preferred not to make an uncritical affiliation to any of these criteria, so we opted for the application of both (i.e., two-sigma and three-sigma rules), and the descriptive study of the filtered data that resulted in each case. For both exclusion criteria, the variable analyzed was the number of actions on the test object; in line with Graham et al., (2013), we conducted the study of outliers only on the basis of critical data. The use of a two-sigma rule was able to exclude significant outliers not identified by the three-sigma rule (six vs. two outliers respectively, the same number in each group.).

### Inferential Statistics

We generated some models through inferential statistics to analyze the data. For the pre-identification of the significant relationships, a hypothesis contrast was carried out among the groups associated with the three main explanatory variables, namely, LABEL (Same, Distinct), CONDITION (Unpredicted, Predicted, Baseline), and SIMILARITY (High, Low), assuming that the dependent variable was the number of actions on the test object.

We did not find any significant differences for the two-sigma rule, or for the three-sigma rule regarding the conditions of interest: Same Label Unpredicted vs Distinct Label Unpredicted. Table 3 shows the contrasts performed for a two-sigma outlier exclusion rule.

**Table 3:** Pairwise comparisons for a two-sigma outlier exclusion rule for the main relevant groups

Cases	Mean number of actions on test object	Target actions on test object	t-value	p-value
All	Same vs Distinct	5.61 vs 4.57	0.879	0.380**
LABEL = “same”	Baseline vs Unpredicted	1.75 vs 6.84	3.50	0.002***
LABEL = “distinct”	Baseline vs Unpredicted	1.38 vs 4.67	2.89	0.007***
CONDITION = “baseline”	Same vs Distinct	1.74 vs 1.38	0.440	0.660
CONDITION = “unpredicted”	Same vs Distinct	6.84 vs 4.67	1.31	0.200**
LABEL = “same label” and CONDITION = “baseline”	High vs Low	2.33 vs 1.20	0.802	0.440
LABEL = “same" and CONDITION = “unpredicted”	High vs Low	5.70 vs 8.11	0.913	0.380
LABEL = “distinct” and CONDITION = “baseline"	High vs Low	1.50 vs 1.25	0.261	0.800
LABEL = “distinct” and CONDITION = “unpredicted”	High vs Low	5.33 vs 4.00	0.634	0.530

Significance codes: 0 ‘***’ .001 ‘**’ .01 ‘*’ .05 ‘.’ .1 ‘’ 1

**Fig. 6:**
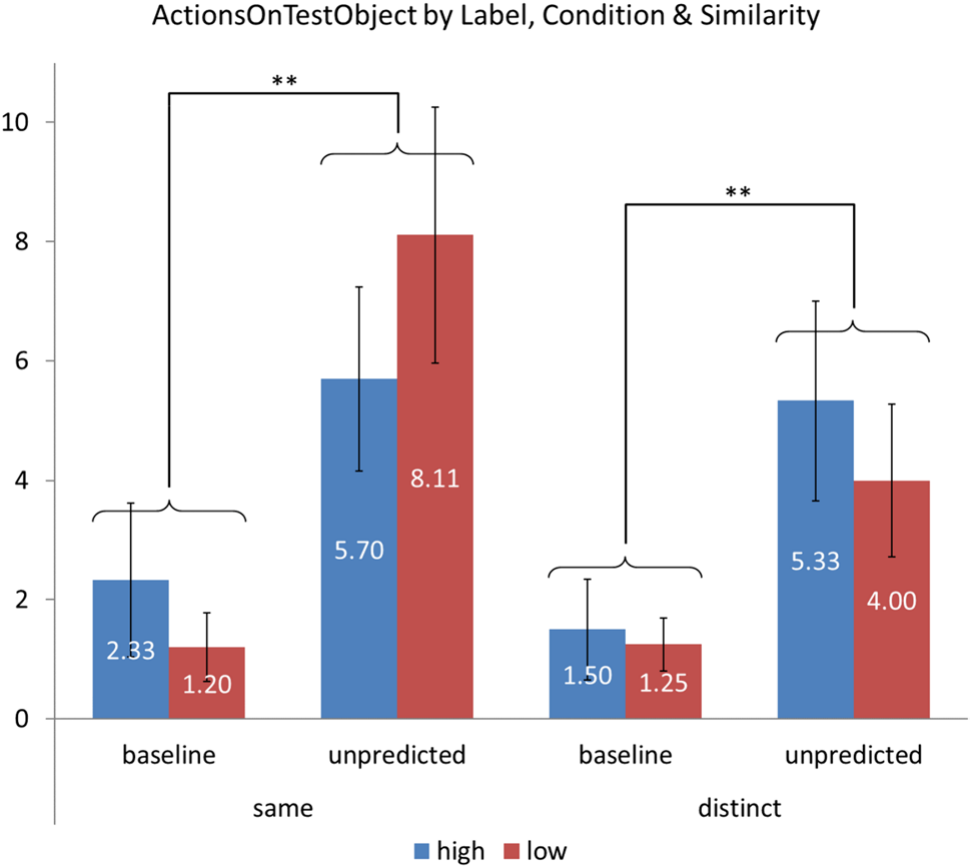
Mean number of target actions performed on test objects as a function of label group, condition, and similarity (two-sigma outlier exclusion rule)

The two pairs of groups in which significant differences were identified were Same Label Unpredicted and Distinct Label Unpredicted versus their respective Baseline conditions. Thus, it was found that, on the one hand, for Same Label, the difference between the Baseline (1.75 actions) and Unpredicted (6.84 actions) was significant (p-value = 0.002), and that the same happened in the case of the Distinct Label for the difference between Baseline (1.38 actions) and Unpredicted (4.67 actions), which was also significant (p-value = 0.007). However, the comparison between the critical groups, with Same Label + Unpredicted (6.84 actions), on the one hand, and Distinct Label + Unpredicted (4.67 actions), on the other, did not show a significant difference between them (p-value = 0.20). All these relationships are shown graphically in Fig. 6.

To complete the characterization of the influence of the main relevant variables, a mixed effects model was run to describe the target variable (i.e., number of actions). This is a departure from Graham et al.'s approach, which used mixed ANOVA models to perform this characterization. We preferred to use mixed-effects models because they allow for more flexibility in specifying the model, allowing for both fixed and random effects, making it possible to analyze the data more comprehensively (Baayen et al., 2008; Barr et al., 2013). In our case, mixed effects models allowed us to account for random effects due to repeated measures within participants and items (i.e., pseudowords) that ANOVAs cannot adequately capture (Yu et al., 2022), thus providing more precise and generalizable results. In any case, Graham's analyses based on mixed ANOVAs were also reproduced in order to compare their results with those of the mixed-effects models.

#### Mixed ANOVA Models

Firstly, we reproduced all Graham et al.'s analyses with mixed ANOVA models, namely: (A) Three-way mixed ANOVA: 2 Label (same-distinct) × 2 Condition (baseline-unpredicted) × 2 Similarity (Low–High) (see Table 4); (B) Two-way mixed ANOVA (for Condition = baseline): 2 Label (Same-Distinct) × 2 Similarity (Low–High) (see Table 5); (C) Two-way mixed ANOVA (for Condition = Unpredicted): 2 Label (Same-Distinct) × 2 Similarity (Low–High) (see Table 6). The models included all possible interactions between the considered explanatory variables. The analyses were performed even though the Shapiro test did not confirm that the data came from normal distributions, since the QQ plots suggested that the distribution might be normal for most of these groups. As a result, we obtained a main effect only for the CONDITION variable in the three-way mixed ANOVA (p < 0.001), with participants in the Same Label group performing more actions than those in the Distinct Label group. No other main effect was found for the remaining variables (i.e., LABEL and SIMILARITY) in the three-way ANOVA model. As expected from the results obtained in the three-way ANOVA models, no main effect was found in the two-way ANOVAs, when the CONDITION variable was used to select the cases examined in each of these two analyses. Finally, the mixed ANOVA models were conducted with the two abovementioned treatments of outliers (i.e., two-sigma and three-sigma rules), and there were no differences between the results obtained in either case.

**Table 4:** Three-way mixed ANOVA: 2 Label (same-distinct) × 2 Condition (baseline-unpredicted) × 2 Similarity (low–high); (for a two-sigma outlier exclusion rule)

Effect	SSn	SSd	F	p value
(Intercept)	1146.5	321.6	67.728	1.1E-07*
Label	41.7	321.6	2.463	0.133
Condition	372.8	400.3	17.695	4.8E-04*
Similarity	0.3	272.5	0.020	0.889
Label: Condition	19.2	400.3	0.910	0.352
Label: Similarity	9.3	272.5	0.651	0.430
Condition: Condition	5.3	396.5	0.253	0.621
Label: Condition: Similarity	22.6	396.5	1.084	0.311

*Note 1:* Significance codes: 0 ‘***’ .001 ‘**’ .01 ‘*’ .05 ‘.’ .1 ‘’ 1

*Note 2:* DFn = 1, DFd = 19 for all effects

**Table 5:** Two-way mixed ANOVA (for CONDITION = baseline): 2 Label (same-distinct) × 2 Similarity (low–high); (for a two-sigma outlier exclusion rule)

Effect	SSn	SSd	F	p value
(Intercept)	105.9	111.1	18.102	4.3E-04*
Label	2.2	111.1	0.369	0.551
Similarity	4.0	158.1	0.483	0.496
Label:Similarity	1.4	158.1	0.174	0.681

*Note 1:* Significance codes: 0 ‘***’ .001 ‘**’ .01 ‘*’ .05 ‘.’ .1 ‘’ 1

*Note 2:* DFn = 1, DFd = 19 for all effects

**Table 6:** Two-way mixed ANOVA (for CONDITION = unpredicted): 2 Label (same-distinct) × 2 Similarity (low–high); (for a two-sigma outlier exclusion rule)

Effect	SSn	SSd	F	p value
(Intercept)	1413.4	610.8	43.967	2.4E-06*
Label	58.7	610.8	1.826	0.192
Similarity	1.6	510.8	0.058	0.812
Label: Similarity	30.5	510.8	1.135	0.300

*Note 1:* Significance codes: 0 ‘***’ .001 ‘**’ .01 ‘*’ .05 ‘.’ .1 ‘’ 1

*Note 2:* DFn = 1, DFd = 19 for all effects

#### Mixed effects Models

In the case of the mixed-effects models, we characterized the target variable (i.e., number of actions on the test object) with a model that included the variables LABEL and CONDITION—as well as their interaction—as fixed factors, and PARTICIPANT and PSEUDOWORD as random effects (in both cases without random slopes). The model had the following form:

LOG_ActionsOnTestObject ~ Label + Condition + Label: Condition + (1|Participant) + (1|Pseudoword).

In the model, the reference levels for the categorical explanatory variables were Distinct (for the LABEL variable) and Unpredicted (for the CONDITION variable). Additionally, our dependent variable ‘LOG_ActionsOnTestObject’ is a logarithmic transformation of the original number of actions on test objects, due to the non-constant variance found in some cases for the response variable. Table 7 shows the estimated coefficients for each variable and their significance for a selection of data according to a 2-sigma outlier exclusion rule. We do not show results for the 3-sigma criterion, since they do not differ in any interesting way from the ones based on the 2-sigma rule.

**Table 7:** Mixed effects model for the variable “number of actions on test object” (for a two-sigma outlier exclusion rule)

	Estimated coefficient	Converted coefficient %	Standard deviation	t-value	Prob.( >|t|)
(Intercept)	1.294	264.7	0.250	5.17	0.0003***
LABEL = "same"	0.470	60.0	0.287	1.64	0.105
CONDITION = "baseline"	− 0.646	− 47.6	0.243	− 2.66	0.009**
CONDITION = "predicted"	0.325	38.4	0.240	1.36	0.177
LABEL = "same" and CONDITION = "baseline"	− 0.556	− 42.7	0.367	− 1.52	0.132
LABEL = "same" and CONDITION = "predicted"	− 0.352	− 29.7	0.361	− 0.97	0.332

*Note 1:* Significance codes: 0 ‘***’ .001 ‘**’ .01 ‘*’ .05 ‘.’ .1 ‘’ 1

*Note 2:* Reference levels: LABEL = "distinct" & CONDITION = "unpredicted"

*Note 3:* Converted coefficient = exp (Estimated coefficient)—1 [expressed as a percentage]

Since the model uses a logarithmic transformation, the meaning of the coefficients requires some explanation, as far as they express the relationship between the explanatory variables and the logarithm of the variable to be explained. In this case, the model coefficients report the following relationships. In the first place, the number of actions on the test object for Unpredicted is 60% greater for Same Label than for the Distinct Label. Secondly, for Distinct Label, the number of actions is 47.6% lower in the Baseline than in the Unpredicted condition, while for Distinct Label it is 38.4% higher in the Predicted than in the Unpredicted condition. Finally, the interactions between LABEL and CONDITION show that, for Same Label, the number of actions is 69.9% lower in Baseline than in Unpredicted, and 2.7% lower in Predicted than in Unpredicted. To sum up, the mixed effects models as well as the ANOVA analyses failed to show a main effect for the LABEL variable.

## Discussion

The goal of this paper was to collect data on autistic children’s sensitivity to the labeling effect, by reproducing Graham et al.,’s (2013) experimental design. Specifically, we hypothesized that we may not find a significant difference between the Unpredicted condition in the Same Label group and the Unpredicted condition in the Distinct Label group, and this is in effect what the results suggest. We summarize and discuss the findings below.

Within the Same Label group, we observed that children imitated the target action significantly more often in the Unpredicted condition than in the Baseline condition. This in principle suggests that there was a violation of the children’s expectations. When the children saw that an object within a pair of same-labeled objects produced a sound, they seemed to expect that the other one would produce the same sound. When it did not, children persisted on an unusual peak of actions that is not present in the Baseline condition. However, we observed the same pattern in the Distinct Label group: children would perform more actions in the Unpredicted condition than in the Baseline condition, which is not what Graham et al. found in their study.

More importantly, the comparison between groups (Same label, Distinct label) in the Unpredicted condition does not support the presence of the labeling effect. When two objects receive the same label, children should expect that they will have the same hidden properties; however, this expectation should be significantly reduced if these objects have different names, even if, like in the experimental setting we proposed, in both cases they are handed one object after the other. A simple visual inspection of Figs. 1 and 6 suggests that the effect, if actual, was much more attenuated in the autistic children in the current study than in Graham et al.’s TD children. However, neither the mixed ANOVA analysis Graham et al. performed nor the analysis using mixed effects models that we also tried supported the view that autistic children are also sensitive to the labeling effect.

Interestingly, as in Graham et al., (2013), we observed that objects with Low similarity elicited a greater number of actions than those with High similarity in the Same Label group (in the Unpredicted Condition). The effect was in fact stronger than the one observed by Graham et al. In the Distinct Label group, we can observe, again as in Graham et al., (2013), that perceptual similarity has more impact on categorization, such that, in the absence of labeling as a possible criterion for a generalization of the category, children base their generalization on the objects’ appearance. However, while this inter-group difference might suggest some sensitivity to labeling (in the sense that labeling may override perceptual similarity), we lack an explanation as to why children in the Same Label group would perform more actions when objects are less similar than when they are more similar.

Our working hypothesis was that autistic children would have a different, diminished or delayed, sensitivity to the labeling effect than TD children of similar age (Gelman & Markman, 1987; Sloutsky & Fisher, 2012). The hypothesis was based on several characteristics of autistic individuals. First of all, the labeling effect is hypothesized to be an invitation to form categories, an invitation that plausibly involves understanding labels as social signals (Ferguson & Waxman, 2017). TD children seem to recognize them as such, and, when it comes to forming categories, to discriminate sounds corresponding to possible words from other sound signals such as tone sequences (Ferry et al., 2010). Autistic children may be less sensitive to this aspect of labels as social signals. Relatedly, the act of naming seems to involve a complexity that, it may be supposed, many autistic children with issues understanding uses of language may not grasp. According to what is observed in the labeling effect in TD children, naming is a speech act that conveys information not only about what name a certain object has, but also about what categories speakers consider there are in the world. This latter constituent of the act of naming may be considered an indirect way of providing information. Grasping this kind of information that is only indirectly conveyed may be delayed in autism. Finally, the labeling effect not only involves generalization, which some studies have found compromised in autistic children, but it also involves revising spontaneous ways of categorizing, which requires cognitive flexibility. At the present stage, we cannot know which one of these characteristics may have had more weight in the results.

## Limitations

The present study aimed to investigate whether there is sensitivity to the labeling effect in autistic children between three and nine years of age. Based on observed socio-communicative and generalization difficulties, we hypothesized that the effect would not be as remarkable as in TD children and that it might not even occur. The data from our study suggest that autistic children are not sensitive to the labeling effect (or at any rate, not as sensitive as TD children are).

To our knowledge, this is the first study aimed at assessing the labeling effect in autistic children. As such, our conclusions should be interpreted with caution because additional research is needed. Some limitations of our study are the following:

- Children displayed a wide within- and between-group disparity in chronological age, and verbal mental age (see Table 1). Regarding between-group similarity, they share the same values in the autism severity mean, but within-group, they are not homogenous in that variable, either.
- The task crucially depends on imitation skills. Although all the children demonstrated to be able to imitate in the warm-up phase, children were not evaluated on imitation skills, and so group formation did not take into consideration how much or how little the children imitated. This may be a minor limitation if, as some suggest (e.g., Zachor et al., 2010), imitation skills relate to autism severity.
- By taking the mean difference between the Same Label and Distinct Label groups as the only criterion for the effect, we could not analyze each case individually, and see how each child reacted to the introduction of labels in the different conditions. Moreover, given the design, we could not observe how the same child would react to the Same Label and the Distinct Label groups.
- As mentioned above, the two groups were matched on autism severity scores. It cannot be discarded that a different way of matching both groups would give rise to a different outcome. Studies with TD children consistently show a labeling effect in children ranging from 10 months to 11 years of (chronological and verbal) age. However, it can be that the level of linguistic development in atypical profiles is related to grasping the implications of labeling. We carried out a post-hoc study with a subset of the sample matched on VMA, which yielded two age groups: children with one and two years of VMA (Group 1, N = 8, four children in the Same Label group and four children in the Distinct Label Group) and children with three and four years of VMA (Group 2, N = 5, three children in Same and two in Distinct). Yet, we did not find significant differences in any of the VMA groups, although the difference between the Same Label and the Distinct label group in the group of three- and four-year-olds in VMA, though not significant, was greater than the one observed in the case of the group of one- and two-year-olds in VMA. Pending further research, this may suggest a delay in sensitivity to the labeling effect in the autistic population.
- We reproduced one experiment whose results are consistent with many others in TD children. However, there may be limitations of the original study we are not aware of, which may also affect our results. On the other hand, the study involves object manipulation and imitation of actions. Perhaps studies employing different methodologies give rise to different outcomes.

## Conclusions

As argued for at the outset of this article, it is important to study the labeling effect in autistic children because the labeling effect makes children adjust their conceptual categories to those of their linguistic communities. As Waxman & Markow, (1995) put it, children accept the invitation to form the categories that language affords. Labels also contribute to consolidate concepts in memory. Both effects related to labeling are of utmost importance in social and cognitive development, and it is therefore certainly relevant to study to what extent the labeling effect can appear diminished or delayed in autistic children. If autistic children exhibit difficulties in inferring that two objects that receive the same name form a category, they may tend to generate concepts that do not match the concepts that their TD peers have formed under the indirect guidance of other members of their linguistic community. This may result not just in linguistic communication difficulties, but also in different ways of thinking about the world. As some authors have noted, the labeling effect has some (at least mild) Whorfian consequences (Henningsen-Schomers et al., 2022; Lupyan, 2012; Vicente & Martinez-Manrique, 2013), which implies that sensitivity to labels impacts habitual ways of thinking, with downstream consequences for communication and mutual understanding. Surprisingly, the labeling effect is understudied in atypical populations in general, and in autism in particular. Work on conceptual development in autism has explored whether autistic children are stricter in the way they generalize (Froehlich et al., 2012; Naigles & Tek, 2017; Wimmer et al., 2023), and work on lexical acquisition has studied whether children generalize labels according to shape, as TD children do (Hartley & Allen, 2014). Generating conceptual categories based on labeling plausibly has a deep impact on conceptual and linguistic developmental trajectories, at least as deep as the just mentioned, and well established, research topics.

Likewise, it is important to include considerations of the labeling effect in intervention programs. Some intervention programs target vocabulary learning and concept building mechanisms specifically, as for instance word learning by exclusion (see e.g. de Marchena et al., 2011; Hartley et al., 2019, for evidence on delay in word learning by exclusion in autism, and Carr, 2003; Sivaraman & Bhabu, 2018, for intervention studies on this word learning exclusion mechanism). On the side of concept building abilities, some intervention programs have targeted generalization from exemplars (Erhard et al., 2021; Soulières et al., 2011), which, as mentioned, has been found to be narrower in autism.

In general, intervention programs should consider devoting more effort to training autistic children in vocabulary and concept building abilities. According to Naigles & Tek, (2017), across the spectrum, most of the difficulties of individuals across the autism spectrum relate to the conceptual-semantic component of language. Lexical entries in autistic minds tend to store narrower and different information than lexical entries in neurotypical minds. However, as explained, another probable source of conceptual misalignment between autistic and neurotypical individuals with downstream consequences for communication, is exhibiting a diminished or delayed sensitivity to the implications of labeling. Programs targeting conceptual generalization should probably include training paradigms focused on teaching such implications of labeling. Autistic children could be exposed to labeling situations like the one exemplified by the experiment we have reported and be reinforced until they start expecting that two objects with the same label will share non-obvious properties (and that objects with different labels do not share them).^9^ If they generate such an expectation and generalize it to other kinds of non-obvious properties (i.e., properties that are not sounds), that will mean that they have become sensitive to the role of labels. Much is still unknown about what effects labeling has in development, but if it is indeed a way to generate stable categories in mind, diminished or delayed sensitivity to labeling should be compensated for with training.

## Footnotes

1 See Laurence & Margolis (1999) for a classical statement of what concepts are and how they support inferences about non-obvious properties.

2 For more, abundant, references, see Graham et al., 2013, p. 1–2.

3 In general, autistic children exhibit difficulties in the area of pragmatics (Schaeffer et al., 2023). Such difficulties include, *inter alia*, learning new words by exclusion — i.e., identifying a referent for a novel word on the basis of excluding other possible referents for which names are known (Hartley et al., 2019). In our view, word learning by exclusion seems to be a simpler process than understanding that two objects labeled in the same way form a category (and therefore have interesting properties in common).

4 According to Henningsen-Schomers et al. (2002), such an effect would especially affect abstract concepts (while also affecting concrete ones).

5 The bar plot describes a comparison between the Baseline and Unpredicted conditions, and the high vs. low similarity conditions. These are not critical to understand the main message of Graham et al.’s study, but we will come back to them in the Methods section, when we provide detail of the design of our study, which follows theirs.

6 The labeling effect has proven to be robust from at least 10 months to at least 11 years of age in typical development. By testing autistic participants within that range of age, we thought we could obtain results suggesting difficulties at that age range. Such difficulties are compatible with a delay, rather than persistent difficulties, in the area of concept generation by labeling. We thank an anonymous reviewer for making this point.

7 All children imitated. Yet, there may be differences in imitation degree among the children. We did not control for imitation abilities when creating the Same Label/ Different Label groups, which may have affected the results. However, note that imitation abilities may relate to autism severity (e.g., Zachor et al., 2010), which was the guiding parameter that we used.

8 We follow Graham et al.’s terminology to avoid misunderstandings, even though we think that Graham et al.’s Distinct Label Unpredicted condition should be rather called “Predicted”, since children do predict that when two objects receive different labels, they do not share non-obvious properties.

9 In fact, some of the more verbal children in our experiment commented that they had realized what the experiment was about, namely, that only the things with the same label made the same sound.
